# p3k14c, a synthetic global database of archaeological radiocarbon dates

**DOI:** 10.1038/s41597-022-01118-7

**Published:** 2022-01-27

**Authors:** Darcy Bird, Lux Miranda, Marc Vander Linden, Erick Robinson, R. Kyle Bocinsky, Chris Nicholson, José M. Capriles, Judson Byrd Finley, Eugenia M. Gayo, Adolfo Gil, Jade d’Alpoim Guedes, Julie A. Hoggarth, Andrea Kay, Emma Loftus, Umberto Lombardo, Madeline Mackie, Alessio Palmisano, Steinar Solheim, Robert L. Kelly, Jacob Freeman

**Affiliations:** 1 Max Planck Institute for the Science of Human History, Jena, Germany; 2 Department of Anthropology, Washington State University, Pullman, USA; 3 Department of Industrial Engineering and Management Systems, University of Central Florida, Orlando, USA; 4 Department of Archaeology and Anthropology, Bournemouth University, Poole, UK; 5 Department of Anthropology, Boise State University, Boise, USA; 6 Montana Climate Office, WA Franke College of Forestry and Conservation, University of Montana, Missoula, USA; 7 Center for Digital Antiquity, School of Human Evolution and Social Change, Arizona State University, Tempe, USA; 8 Department of Anthropology, The Pennsylvania State University, State College, USA; 9 Anthropology Program, Utah State University, Logan, USA; 10 Center of Applied Ecology and Sustainability (CAPES) & Nucleo Milenio UPWELL, Santiago, Chile; 11 Instituto de Evolución, Ecología Histórica y Ambiente (CONICET & UTN), Mendoza, Argentina; 12 Department of Anthropology, Scripps Institution of Oceanography, University of California - San Diego, San Diego, USA; 13 Department of Anthropology & Institute of Archaeology, Baylor University, Waco, USA; 14 Department of Archaeology, University of Cambridge, Cambridge, UK; 15 Institute of Geography, University of Bern, Bern, Switzerland; 16 Department of Sociology and Anthropology, Weber State University, Ogden, USA; 17 Department of Ancient History, Ludwig-Maximilians-Universität München, München, Germany; 18 Museum of Cultural History, University of Oslo, Oslo, Norway; 19 Department of Anthropology, University of Wyoming, Laramie, USA; 20 The Ecology Center, Utah State University, Logan, USA

**Keywords:** Archaeology, Chemistry

## Abstract

Archaeologists increasingly use large radiocarbon databases to model prehistoric human demography (also termed paleo-demography). Numerous independent projects, funded over the past decade, have assembled such databases from multiple regions of the world. These data provide unprecedented potential for comparative research on human population ecology and the evolution of social-ecological systems across the Earth. However, these databases have been developed using different sample selection criteria, which has resulted in interoperability issues for global-scale, comparative paleo-demographic research and integration with paleoclimate and paleoenvironmental data. We present a synthetic, global-scale archaeological radiocarbon database composed of 180,070 radiocarbon dates that have been cleaned according to a standardized sample selection criteria. This database increases the reusability of archaeological radiocarbon data and streamlines quality control assessments for various types of paleo-demographic research. As part of an assessment of data quality, we conduct two analyses of sampling bias in the global database at multiple scales. This database is ideal for paleo-demographic research focused on dates-as-data, bayesian modeling, or summed probability distribution methodologies.

## Abstract (summary)

**Table:** 

Measurement(s)	radiocarbon age
Technology Type(s)	accelerator mass spectrometer
Sample Characteristic - Location	global

Machine-accessible metadata file describing the reported data: 10.6084/m9.figshare.15152097

## Background & Summary

The interaction between human population growth and the sustainability of the Earth’s life support systems is of central interest for interdisciplinary science, the public, and policymakers. Yet, the causes of long-term population increase or decline are still not well understood. In part, this is because of a lack of data relevant for studying long-term changes in human populations. Archaeological records provide unprecedented time-depth for research on the drivers of human population growth and the impacts this growth has had on ecosystems. Radiocarbon data provides the most temporally and spatially widespread archaeological data available for paleodemography and human population ecology research. The link between radiocarbon and population is straightforward: Human activity produces organic waste as humans consume energy (cook, clean, farm), which has a robust sublinear relationship with human population size^1^. Archaeologists sample these organic waste products from their archaeological sites to date events in prehistory. Paleodemographers can, thus, compile radiocarbon data to identify general trends in human activity and infer changes in population in their research area^2,3^. For this reason, the past two decades have witnessed an explosion in the development of archaeological radiocarbon datasets from various regions of the world. These datasets have great potential for comparative global-scale research on human population growth and ecology throughout the Holocene (e.g.^4–7^).

However, the potential of archaeological radiocarbon datasets for globally-comparative research on human paleo-demography, at present, faces challenges. These challenges center on the issue of FAIR (findable, accessible, interoperable, reusable) principles in scientific database management and stewardship^8^. First, archaeological radiocarbon datasets are currently held in a variety of different repositories (e.g., CARD, tDAR, INQUA, among others) that limit their findability and accessibility. Second, different data collection protocols have been carried out by the various research teams assembling these datasets, which have generated datasets differing in the quality of their individual samples, and the relative spatial and temporal coverage of these datasets. These differences in data collection protocols limit the interoperability and reproducibility of archaeological radiocarbon datasets for globally-comparative research. These FAIR principle limitations became apparent during the development of the PAGES People3000 (PalEOclimate and the PeopLing of the Earth) working group, which is one of the first international working groups devoted to comparative research on Holocene human population growth and the evolution of different social-ecological systems throughout the world. Global comparisons of human paleo-demography require a synthetic archaeological radiocarbon dataset developed under a unified data selection protocol and deposited in an easily accessible repository.

This data descriptor presents version 1.0.0 of the PAGES People3000 Archaeological Radiocarbon Database (p3k14c) (see Data Availability Section for repositories). It describes the methods used to develop the database, such as the different parent databases from which the database was synthesized, the criteria involved in the unified data collection protocol, and the computational methods used to clean and organize these data according to this protocol. In addition, this data descriptor presents frequency statistical summaries of the different types of radiocarbon samples and their relative quality for each region included in the dataset. Spatial analyses are carried out in order to present the different quality of these data by region, which provides an assessment of which regions have less biased, and therefore more robust datasets, and which regions need further investigation and more caution in comparative analyses.

The data are useful for a range of different comparative analyses, not only of human paleo-demography, but also the evolution of energy consumption by different social-ecological systems and global to regional-scale models of human land-use intensification and change^9–12^. In addition, the data are useful for considerations of cultural heritage management, such as regions of the world threatened by a range of different impacts brought about by climate changes (sea-level rise, ice-patch melting, desertification)^13^. Lastly, this dataset can be integrated with paleo-climate and paleo-environmental data from around the world to examine the extent and complexities in the relationships between human population, climate conditions, and resource availability at multiple scales. In the remainder of this paper, we describe the data and analysis aimed to understand data quality and biases so that researchers can use the data in an informed fashion.

## Methods

The p3k14c database comprises a total of 180,070 archaeological radiocarbon ages from around the world. This database is composed of data from previously published integrated databases, as well as data from publications not currently included in extant databases. In this section, we discuss the methodological needs for large radiocarbon datasets, especially chronometric hygiene methods, as well as quality control analysis, and requirements for published radiocarbon databases. These steps are necessary from a methodological and ethical standpoint when analysing large radiocarbon databases. Given the different radiocarbon collection standards throughout the world, our quality analysis provides insight into the spatio-temporal regions for which radiocarbon should be an effective way to study paleodemography, especially long-term growth processes, and those regions with clear biases in the records.

### Procedures — data sources

The p3k14c database combines, verifies, and cleans data from 39 radiocarbon sources (Table 1), including data published as part of research articles and data gathered exclusively for database formation. Some were freely available already, while others were available upon request. Of those available upon request, the authors gave us permission to publish their data in this revised format. Given the variability in radiocarbon dataset publication and the tendency to pull from pre-existing datasets, we gathered information listed by the publication or website about where the data came from. In Table 1, the “Parent Dataset(s)” column has two options for “None”: “N/A” means that none are likely given the nature of the dataset, whereas “None listed” means that the compilers may have combined data from pre-existing compilations, but they were not explicit about where published radiocarbon ages came from.

**Table 1:** Database/Dataset name, base LocAccuracy variable, and other relevant information collected.

Database Name	Pub Year	Base LocAccuracy (see Table 2)	Parent Dataset(s) (Note that this is not complete)	Citation
14CARHU	2015	0 or 1	N/A	^65^
14SEA	2017	0	None listed	^45^
aDRAC	2016	1	None listed	^97^
Andes14C	2021	2	Ziolkowski_*et_al*_1994^85^	^90^
AustArch	2014	2	N/A	^98^
Bevan2017	2017	1	ORAU^46^ RADON^47^ EUROEVOL^66^ CalPal^42^ Chapple 2019^99^	^67^
CALPAL	2016	1	N/A	^42^
Capriles_&_Albarracin-Jordan_2013	2013	2	N/A	^91^
CARD	2019	1	None listed	^22^
CONTEXT	2006	1	Website not maintained	^48^
Cremaetal2016	2016	2	N/A	^64^
EUROEVOL	2016	2	RADON^47^ BANADORA^49^	^66^
Flohretal2016	2016	1	CalPal^42^ CONTEXT^48^ Ex Orient^100^	^50^
Gayo_CentralChile	2019	2	SCAR Campbell & Quiroz 2015^101^	^86^
Goldberg_2016	2016	1	SCAR Andes14C^90^ Mendez2013^102^ Bueno *et al*. 2013^103^ Prates *et al*. 2013^104^ Mendez *et al*. 2015^87^ Rademaker *et al*. (2013)^105^ Steele & Politis 2009^106^	^88^
GuedesBocinsky2018#	2018	2	Wangetal2014^61^	^17^
Jorgensen_2020	2016	0	N/A	^68^
Kay_WestAfrica	2019	1 or 2	N/A	^38^
KITEeastafrica	2016	2	N/A	^43^
Lombardo_2020	2020	3	Caprilesetal2019^107^	^92^
ManningTimpson2014	2014	2	Vernet & Aumassip 1992^108^	^40^
MedAfriCarbon	2020	1 through 3	CalPal^42^	^41^
Mendez2013	2013	0	N/A	^102^
Mendezetal2015	2015	1	N/A	^87^
MesoRAD2020	2020	1	N/A	^78^
Palmisano2017_Italy	2017	1 through 3	CalPal^42^ RADON^47^ EUROEVOL^66^ ORAU^46^ IRPA/KIK^109^	^69^
Pratesetal2020#	2020	1	Pratesetal2013^104^	^93^
RADON	2012	2	N/A	^47^
RADON-B	2014	1	None listed	^70^
RapaNui2020	2020	2	Mulrooney2013^110^	^111^
RirisArroyoKalin2019	2019	2	SCAR^112^ Andes14C^90^ Goldberg2016^88^ Bueno *et al*. 2013^103^ Prates *et al*. 2013^104^ Mendez *et al*. 2015^87^ Steele & Politis 2009^106^	^89^
SARD	2019	2	N/A	^44^
SCAR	2015	2	Andes 14C^90^ Rademaker *et al*. 2013^105^	^112^
Silva_VanderLinden_2017	2017	2	Flohretal2016^50^ EUROEVOL^66^	^71^
Solheim_Norway	2018	2	N/A	^72^
UWyo2021	2011	1	N/A	
Vermeersch2019*	2019	1	CalPal^42^	^73^
Wangetal2014	2014	1	N/A	^61^
Ziolkowski_*et_al*_1994	1994	2	N/A	^85^

*The Vermeersch dataset was added last. Only radiocarbon lab numbers NOT already present in the dataset were added to the raw, uncleaned dataset.

^#^These datasets did not have Country provided as a variable, but Country was acquired by plotting the geographic coordinates and conducting a spatial join with the Natural Earth countries shapefile.

These are the only databases listed in the “Source” column.

Some of the datasets that we combine were published, but a larger version was acquired from the authors themselves: the “Pub Year” refers to the dataset rather than the affiliated publication. The “Base LocAccuracy” variable was generated based on the assessed locational accuracy of the data, with 0 referring to “no locational information” while 3 refers to “exact locational information.” Note that most radiocarbon data have a LocAccuracy of 1 or 2 (see Table 2), which are useful for large scale coarse grained analyses, but could not be used to, for example, address fine grained questions such as whether prehistoric populations preferred a particular elevation for settlement in a mountainous environment^14^.

**Table 2:** Database variable names, descriptions, and whether or not the variable was required to include the data.

Variable Name	Required?	Description
LabID	Yes	Unique lab identification for every radiocarbon date
Age	Yes	Radiocarbon age
Error	Yes	One sigma standard error of the radiocarbon age
Material	No	Taken straight from the dataset, no consolidation of materials.
Taxa	No	Taken straight from the dataset if they had a separate column. Again, no cleaning or verification process.
d13C	No	δ^13^C value taken straight from the dataset with no cleaning or verification process. Note that there may be many inaccurate “0” values taken from the original dataset, since several datasets used “0” instead of NA or a blank cell.
Method	No	Refers to method of radiocarbon dating used, such as AMS or radiometric. Taken straight from the dataset with no cleaning or verification process.
Period	No	Archaeological time period. Did not clean or organize. Common in European datasets, generally hit-or-miss elsewhere.
SiteID	No	Site identification number. Very useful for US and Canadian sites, otherwise uncommon.
SiteName	No	Site name, usually unique to each site within each country. Common in non-North American sites
Long	No	Longitude, preferably in decimal degrees, but degrees, minutes, and seconds also accepted. Any other format was excluded.
Lat	No	Latitude, preferably in decimal degrees, but degrees, minutes, and seconds also accepted. Any other format was excluded.
LocAccuracy	Yes	Variable created according to each dataset’s described accuracy and verified later. Necessary to prioritize radiocarbon ages that came from more reliable sources (e.g. directly from collector) 0: no specific locational information: only country provided 1: Province/State (not-US) or county (US) locational information. Note that the accuracy varies according to how large the country, province, and counties are. 2: Very close locational information (within 500 m), including locations digitized from forms and found during internet search 3: Exact location of site provided. Source collected location personally.
Country	No	The country listed or provided by a dataset affiliated with the date and verified later. If no country was provided by the dataset, the data were retained but lat/long were not verified except to ensure they were on the appropriate continent.
Province	No	Administrative province or state within a country
Region	No	Variable generated according to country and province, if available. A broader region of the world.
Continent	Yes	Provided by dataset and verified later. Any dates without an affiliated continent (or one that could be determined according to a listed country) were deleted.
Source	Yes	The dataset that provided the date.
Reference	No	Full reference if available, but short (e.g. author and year) reference also accepted for the radiocarbon date information. Provided by dataset. No verification process, but extra whitespace removed.

Note that many variables are not required but are very useful.

Once these datasets were gathered, only data labelled as archaeological dates (not, for example, geological or paleoecological dates) in the original publication (or dataset) were extracted. We did not systematically check for consistency between the actual data and its label in the original publication. These data were not individually verified for archaeological affiliation: we assumed the dataset compilers and submitters did a reasonable job of ensuring their local archaeological datasets had only archaeological data. As such, there are some data that may appear erroneously old to some (e.g., AECV-1581C, is 35500 ± 2530 14 C BP, is identified as “wood” from Canada). We recommend researchers select data that are appropriate for their research and conduct independent verification (i.e. via original site reports or radiocarbon sample publications) as necessary for very early or seemingly erroneous dates.

### Procedures — data selection

Within our large dataset, we collected information important for using large data sets of radiocarbon ages to estimate human population processes (Table 2). The three most important variables are lab number, radiocarbon age, and one sigma standard error for a valid radiocarbon age. Note that we gathered normalized radiocarbon ages if available, measured radiocarbon ages if not. We did not convert the measured radiocarbon ages to normalized radiocarbon ages, though we did gather δ^13^C values if available. We also gathered material and taxa information from the original dataset. Thus, while present, these data are not uniform as the researchers who put together the various component data sets do not use the same language. We gathered archaeological time period data if available: this column is common in European datasets. Again, we did not ensure uniformity or language. We gathered SiteID (common in US and Canada) and SiteName (common outside US and Canada): we assumed the submitters properly spelled these in their original language. We gathered latitude and longitude, which are necessary for the LocAccuracy variable (mentioned previously, more detail in Table 2), as well as Country, Province, and Continent. We standardized the Country column to match modern naming conventions (e.g., Macedonia, FYROM, and North Macedonia were all standardized to North Macedonia). We attempted to remain apolitical regarding disputed territories by maintaining country names according to what was provided in the original dataset. We recognize that political boundaries can be contentious, and we made no deliberate slights. Finally, we used Country designations to produce Region designations to allow the consideration of dates without Lat/Long (LocAccuracy 0).

We assigned LocAccuracy values for all radiocarbon ages with Lat/Long coordinates according to the gathering methodology listed in the affiliated document or personal communication. Most LocAccuracy values were assigned as “1” if they had minimal information or if the methods support this designation while “2” and “3” were assigned only when the authors described sufficiently precise gathering criteria. Finally, we gathered the reference listed in the dataset and the nickname of the dataset itself (see Table 1 for abbreviations and citations). Once these data were gathered into one large dataset, we began to clean the dataset, first manually, then automatically.

### Procedures — manual cleaning

**Fig. 1:**
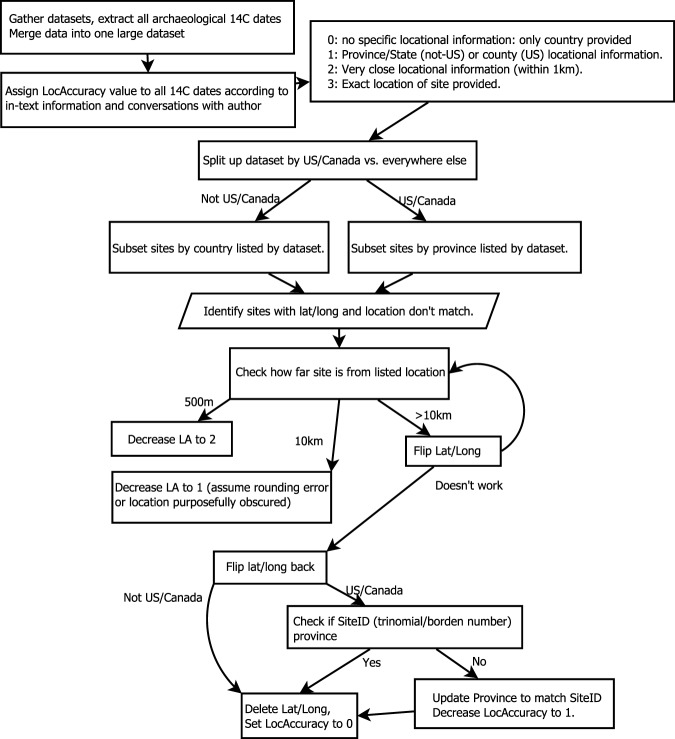
Flowchart demonstrating the decision-making process for verifying the location and modifying the LocAccuracy variable accordingly.

After removing clearly geological or paleontological ages, we had 272,534 radiocarbon ages that needed to be verified in a rigorous, consistent way (Fig. 1). The goal of this manual cleaning phase is to verify the LocAccuracy variable so that the most accurate information is retained during the course of the automated cleaning phase. The data were divided according to continent, with additional steps taken for the U.S. and Canada dates. All dates with locational information were compared with Natural Earth’s Admin 0 – Countries^15^ layer or, in the case of the U.S. and Canada, Natural Earth’s Admin 1 – States, Provinces^16^ layer. These data were compared in QGIS3 using the “Vector/Data Management Tools/Join Attributes by Location…” tool. Any mismatched locations were manually inspected using the following methodology.

First, the distance between the lat/long location and the listed country were compared (Fig. 1). If the location was within 500 m of the listed country/province, we decreased LocAccuracy to 2 with the assumption this was a rounding, purposeful blurring of location, or a GPS error. If it was within 10 km, LocAccuracy was decreased to 1 for the same reasons. If it is greater than 10 km from the listed location, we then flipped the coordinates so that latitude became longitude and vice versa to check for input error. We then tested these “new” coordinates against the listed location, repeating the first step. We then had one more available step with the U.S. and Canada dates: we could use the SiteID for additional information, if available. In these countries, SiteID is assigned according to state (U.S.) or location within a block grid (Canada). For those radiocarbon ages with SiteID’s, we updated the Province to match the SiteID’s affiliated province and decreased the LocAccuracy to 1. Otherwise, we deleted the values in the Latitude and Longitude columns and decreased LocAccuracy to 0.

There are some political holdovers we considered. Some sites were listed as being in Russia, but the locational information placed them in ex-Soviet countries. Yugoslavia and Czechoslovakia were also sometimes included in the original datasets. In these cases, priority was given to locational coordinates and the Country variable was updated accordingly.

**Fig. 2:**
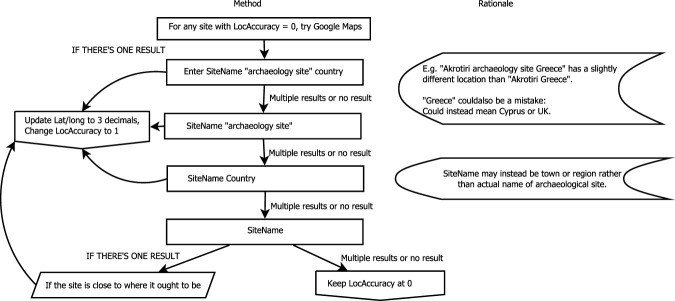
Flowchart used to locate archaeological sites without locational information. Note this method worked best for famous sites.

Once these LocAccuracy assignments were made, we then took additional steps to improve the quality of the data. For any site with a LocAccuracy of 0, we used google maps to check for potential locations (see Fig. 2). We entered in a series of queries and if there resulted in only one location, we updated the latitude and longitude columns with 3 decimals (and verified this abbreviated location would be within the province of the site) and changed the LocAccuracy to 1. We first entered the SiteName, “archaeology site,” and the country. If there were many results or no results, we moved to the next query: the SiteName and “archaeology site,” then SiteName and Country. Finally, sometimes entering just the SiteName provided a result that was actually geographically close to the locational coordinates and the country, in which case we updated the location. If none of these methods worked, we kept LocAccuracy at 0. In the final uncleaned version of the dataset, there are 12,983 radiocarbon ages with a LocAccuracy of 0.

### Preliminary cleaning and dataset review

At this point, we ran the automated procedure described below in the Procedures — Automated Cleaning section. The regional specialists among the co-authors reviewed the product to ensure coarse-grained accuracy of data and minimizing unnecessary deletion of data we knew to be good. Each contributor chose the best method for their particular region of interest. In most cases, this led to the inclusion of more accurate locations and refined radiocarbon lab abbreviations. We provide these manually-cleaned data as **p3k14c_raw.csv**.

### Procedures — automated cleaning (“scrubbing”)

After manual cleaning and data refinement, we developed an automated process in Python to further clean the raw radiocarbon dataset by removing erroneous records; we call this the “scrubbing” process. The process can be broadly defined by five major steps in the following sequence:

1. Removing records with lab codes from unknown laboratories;
2. Standardizing coordinate formats among records with location data;
3. Handling duplicate entries;
4. Miscellaneous cleaning of anomalous data; and
5. Obfuscation of precise coordinates for dates in the United States and Canada, as well as from the^17^ dataset, in order to protect site locations.

Whenever a particular record is removed from the dataset for any reason, a copy of it is stored in a file along with a reason for its removal. This not only creates a detailed record for reasons of posterity and quality assurance, but also allows data creators to understand why their records did not make it into the final dataset so that they might adapt their current data curation practices. It is possible the automated procedure deleted valid radiocarbon dates, in which case we encourage researchers to come forward with corrections. We provide these deleted data as **p3k14c_graveyard.csv**.

Below, we provide a detailed overview of each of these steps. Additionally, we outline a procedure for correcting Unicode encoding anomalies that were present in much of the data.

### Purging records with lab codes from unknown laboratories

In order to verify the veracity of each record, we must ensure that they originate from legitimate sources. We maintain a list of over 400 known laboratory codes in conjunction with the full name of each laboratory and the country it is located in. The laboratory ID (variable LabID) for each record is cross-checked with this list of known codes, and any given record with a LabID that is not in the list of known codes is expunged from the dataset.

### Standardizing coordinate formats among records with location data

Some records provide their location data in a form such as degrees/minutes/seconds or a Northing/Easting coordinate pair. During this step of cleaning, we iterate over each location datum, detect its format, and convert it into latitude/longitude decimal degree format if it is not already so.

### Handling duplicate entries

We define “duplicate” records as multiple records bearing the same LabID. This step is perhaps the most involved step of the cleaning process, as duplicates must be carefully handled to account for a variety of situations:

- If duplicate records have identical ages and identical location data, we arbitrarily keep one duplicate and delete the others, as these are “true duplicates.” For these, each source dataset the record appears in is concatenated into a list for the Source column.
- For records appearing in both the UWyo2021 and CARD datasets, information from UWyo2021 is prioritized since their data gathering process was more systematic.
- If duplicate records have mismatching information for the record’s age, all instances of this record are deleted (as there is no way to determine which age may be the correct one).
- If duplicate records have different LocAccuracy variables, the record with the highest LocAccuracy is always chosen.
- Among variables with identical LocAccuracy, δ13C information within the range of [-30, -1] receives first priority, nonzero values outside this range receive second priority, and non-blank zero values receive third priority.
- Lastly, if duplicate records have identical LocAccuracy but have location data that is too dissimilar (with acceptable dissimilarity defined as ±0.5 degree “fuzz factor” difference in longitude and latitude), we delete all instances of the record.

### Miscellaneous cleaning of anomalous data

The following list details some final minor adjustments to the dataset:

- Records with no information about their age are deleted.
- Records with non-integer ages are deleted; these are anomalous records since radiocarbon dating cannot provide accurate data on such a fine-grained timescale.
- Records with dates from the future (0 or a negative number) are deleted.
- Records with either too large or unreasonably small uncertainty in their age are deleted. We allow records with a minimum error of 15 years. We delete any records with an error larger than its age.
- We delete all records older than 55,000 years, as this is the maximum age for the radiocarbon calibration curve^18^.
- Records with LabIDs containing corrupted unicode characters are removed.
- A handful of records with inconsistent location information that was not automatically detected during the location-standardization step have their location info manually deleted.
- Lastly, some CSV-handling programs (such as the backend for tDAR) are especially sensitive to the handling of quotation marks. Quotation marks are ordinarily used in CSVs to demarcate string values containing characters that otherwise would be interpreted as delimiters (principally, commas). To circumvent the issue of misplaced quotations accidentally merging multiple entries of data, we remove all quotation marks and commas from the datasets’ string entries.

### Obfuscation of precise coordinates for certain dates

In keeping with FAIR Principles recommendation for data to be “as open as possible, as closed as necessary,” we have chosen to obfuscate radiocarbon date locations for the USA, Canada, and from the Guedes and Bocinsky database^17^.

To maintain legal compliance within the United States, under Section 9 of the Archeological Resources Protection Act of 1979, and both the CARD (the only database for Canada) and the Guedes and Bocinsky databases, which are password-protected contributing databases, we do not publicly share precise coordinate locations for archaeological finds. Rather, each coordinate is transformed to be the centroid of the administrative subdivision in which the date is located. This sufficiently obfuscates the data while still allowing for broad, coarse-grained analysis over large regions. This allows us to develop a publicly accessible dataset and at the same time control access to protect archaeological sites from vandalism and looting while maintaining an “open as possible, and closed as necessary” approach to data availability^19,20^.

To accomplish this, we use the Admin2 shapefiles from the GeoBoundaries global database for administrative boundaries^21^. For each date, we first select which subset of shapefiles to use based on either its indicated *Country* info (for US and Canada dates) or its indicated *Source* info (for dates in GuedesBocinsky2018). Then, for each date, we compute which boundary in which it is located and reassign its locational info to that boundary’s centroid. For dates that are very near borders and coastlines and not technically within any of the defined boundaries, we simply choose the boundary that it is closest to in deciding which centroid to use. Should researchers require more precise locations than county/division centroids, the data may still be obtained by reaching out to the relevant State Historic Preservation Office (SHPO) for the UWyo2021 database, on the CARD website for the CARD database, or on tDAR for^17^.

We did not obfuscate the locations for data from other regions of the world, since they are available through unrestricted sources.

### Correction procedure for unicode encoding anomalies

One issue which we encountered while working with the data was large amounts of character encoding errors. Namely, many site names that include characters outside of the standard Latin alphabet became corrupted sometime before we received the raw data (e.g., Alsónémedi was corrupted into Alsónémedi, Barkåker was corrupted into Bark√•ker). This occurs when data curators do not preserve a full Unicode encoding for their datasets. Programs such as Microsoft Excel will often default to the encoding associated with the host machine’s locality (e.g., Windows-1252 encoding for a United States English installation), and thus simply pressing “Save” in such a program can have unintended consequences that can be difficult to detect or correct. Indeed, encoding errors such as this can be found in a variety of already-published radiocarbon datasets such as CARD^22^.

Although there exist a variety of solutions for fixing encoding errors like this (such as the **ftfy** Python module^23^) we found that the whole of our data contained too diverse a variety of different kinds of encoding errors for the solution to be resolved automatically.

To address this issue, we created a specially-suited Python script to manually correct each SiteName encoding anomaly with partial automated assistance. The script detects each encoding anomaly, presents the user with the anomaly within its context, and suggests a variety of possible corrections. The user may then either select one of these suggestions or manually enter in a correction.

This was accomplished by creating a corpus of all uncorrupted words within the raw source radiocarbon datasets, combining it with the GeoNames open-access dataset^24^ of all world cities and regions with a population greater than 500, and feeding this corpus into the SymSpellPy spellchecking correction engine (https://github.com/mammothb/symspellpy). The SymSpell correction engine is then queried with each word containing encoding anomalies and procures a list of suggestions in the corpus within an edit distance of 5.

We found that utilizing this tool, in combination with careful cross-referencing of source datasets and mentions of site names in published scholarly works, was sufficient to enable a 99% success rate for manually fixing each encoding anomaly in the SiteName field.

Additional encoding anomalies persist in the non-machine-interpretable Material and Reference fields. These fields are difficult to fix, given that a record’s material cannot be inferred from the other information in the record (as can be accomplished for the SiteName field utilizing the record’s other locational information), and Reference info is impossible to provide automated suggestions for individual anomalies due to the diversity of spelling possibilities for authors’ names. We have found that the only reliable way to fix these errors is to individually track down the original publication which each record appears in. Given the immense amount of labor that would be required to do this for the entire dataset, we find it reasonable to place the onus onto the end-user for interpreting Material and Reference fields with corrupted characters should they be necessary for a particular project using this database.

## Data Records

Final cleaned radiocarbon data are provided as a single comma-separated values (CSV) file^25^. We will also make available the scrubbed data^26^ and the deleted data^27^, which includes the reason each entry was removed from the cleaned dataset. Additionally, we provide the pre-automated cleaning dataset^28^ (see Table 3 for summary of all provided datasets). Original datasets are all available through their original source (see Table 1).

**Table 3:** Data records and availability information.

Filename	Description	Access	Citation
p3k14c_raw.csv	Manually cleaned raw radiocarbon dataset	Restricted	^28^
p3k14c_scrubbed.csv	Scrubbed radiocarbon dataset without location obfuscation	Restricted	^26^
p3k14c_scrubbed_fuzzed.csv	Final radiocarbon dataset, location information obscured	Public	^25^
p3k14c_graveyard.csv	Data removed from the final dataset during the scrubbing process, including the rationale for removal	Restricted	^27^

The canonical p3k14c data are archived with the Digital Archaeological Record (tDAR) as part of the p3k14c data collection available at https://core.tdar.org/collection/70213/p3k14c-data. tDAR is an archaeological domain repository that specializes in the long-term preservation, access, and reuse of digital archaeological resources.

Within tDAR, there are multiple access levels for datasets that restrict who may download the resource: public, embargo, or confidential (https://www.tdar.org/using-tdar/creating-and-editing-resources/datasets-create-and-edit/). Three datasets contain sensitive site location information — p3k14c Graveyard, p3k14c Scrubbed, p3k14c Raw — and are marked as confidential resources. Confidential resources in tDAR typically contain sensitive data that could endanger an archaeological resource, information that affiliated communities or other interested communities might not wish to be widely available, or information that contributors are not prepared to share. These three datasets can be accessed via a request through tDAR to the resource owner and will remain restricted to professional archeologists and others who will treat the information with proper respect. tDAR data curators maintain long term access to restricted data in case the resource owner is unavailable to provide access, as documented in tDAR’s Preservation and Curation Policy (https://www.tdar.org/about/policies/preservation-and-curation-policy/).

The metadata for the datasets is also available on tDAR.

### Database sustainability

We ultimately aim to turn p3k14c into a fully sustainable ‘living database’ in tDAR by developing a cyber-infrastructure allowing easy uploading of new radiocarbon ages. This cyber-infrastructure develops a means for integrating radiocarbon ages with their associated site reports, which will enhance the ability for critical assessments of sample provenience and lead to more robust multiple proxy approaches to paleo-demography. This proposed cyber-infrastructure also enables archaeological radiocarbon data to be integrated and quantitatively assessed with paleoclimate and paleoenvironmental data. We are confident that this easily usable and integrative cyber-infrastructure will provide a new incentive for producers of archaeological radiocarbon data to continuously contribute their new data.

## Technical Validation

### Basic results of data cleaning

**Fig. 3:**
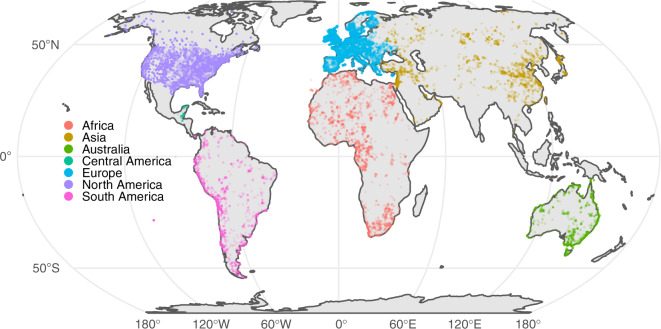
Global map showing locations of all radiocarbon records after the data cleaning process, color-coded by continent. Individual sites are translucent to illustrate site density.

Following the cleaning procedures, our dataset contained 180,070 radiocarbon records (Fig. 3). Results on a continental scale can be found in Table 4. This table refers to the number of records before and after the automated cleaning process, not prior to the manual process. A “dated site” refers to the number of unique SiteName, SiteID, Latitude, and Longitude combinations within each continent. The “Mean Dates / Site” field allows for a quick assessment of sampling quality by dividing the number of Scrubbed Dates by the number of Dated Sites.

**Table 4:** Number of cleaned radiocarbon dates by continent.

Continent	Raw Dates (including duplicates)	Scrubbed Dates	% Kept	Dated Sites	Mean Dates/Site
Africa	14,860	11,129	74.9	3,463	3.21
Asia	21,828	14,071	64.5	2,693	5.23
Australia	3,661	3,657	99.9	1,530	2.39
Europe	119,106	77,393	65.0	21,331	3.63
North America	102,288	64,934	63.5	16,120	4.03
Central America	1,223	1,218	99.5	99	12.3
South America	9,568	7,668	80.1	2,077	3.69
Total	272,534	180,070	66.1	47,313	3.81

### Kernel density analysis

As already pointed out, the database presents major spatial discrepancies in terms of amount and distribution of data. Such biases are evident when considering the dataset in its entirety, but are also shaping the record at smaller, regional scales. While a qualitative assessment of each regional sequence is therefore of paramount importance (see below), it is also possible to account for some of these biases in an explicit quantitative framework. For instance, different traditions of research do not only impact the frequency of dated sites, but also of the intensity of on-site sampling (i.e., the number of dates available for each individual site).

**Fig. 4:**
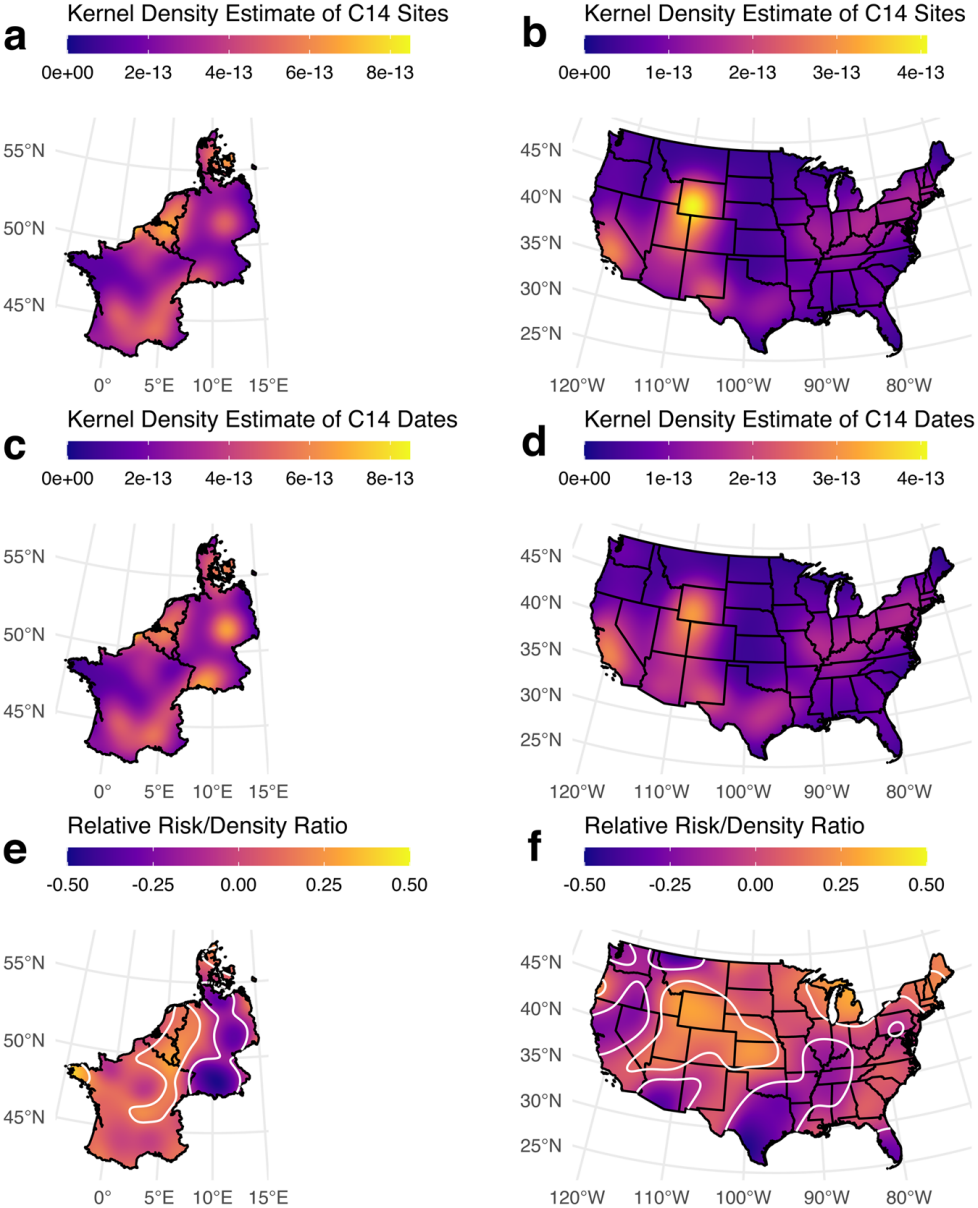
Kernel density estimate highlighting clustering and gaps in different regional/continental records. (**a**,**b**) Kernel density estimates for (**a**) North-Western continental Europe and (B) the Contiguous United States of America. (**c**,**d**) Kernel density estimates weighted by the number of 14C dates per site for (**c**) North-Western continental Europe and (**d**) for the Contiguous United States of America. (**e**,**f**) Risk surface analysis for (**e**) North-Western continental Europe and for (**f**) the Contiguous United States of America. North-Western continental Europe shows significant oversampling of dates in Belgium, the Netherlands, and portions of eastern France, and undersampling across much of eastern Germany; the Contiguous United States shows oversampling across the Great Basin, central Rocky Mountain, central Plains, and New England regions, and undersampling across the Northwest and northern California, southern Southwest, Texas, the American Bottom, and southern Florida regions.

In order to assess the regional role of such practices, we use two-dimensional kernel-density estimates to quantify and visualise spatial sampling bias in the dataset^29,30^. We first computed kernel-density estimates for the distribution of sites (Fig. 4a), and then weighted these by the number of dates available per site (Fig. 4c,d). The comparison of both outcomes was then undertaken by risk surface analysis (i.e., ratio of one two-dimensional kernel-density to another) (Fig. 4e,f)^31,32^. We present here two regional case-studies: continental north-western Europe, corresponding to modern-day countries of France, Belgium, Luxembourg, Netherlands, Germany and Denmark and the continental United States. Both areas were chosen as they present a high coverage of 14 C dates, so that any identified biases represent varying density of sampling, rather than mere absence in the record as often highlighted in criticisms of 14C-based surveys (e.g.^33^).

As can be seen on Fig. 4, although the record in continental north-western Europe is spatially extensive and intensive, the risk surface analysis points to various areas where the kernel density estimates weighted by number of 14 C dates differs significantly from the distribution of dated sites, especially across northern France, Belgium and the Netherlands, areas which have indeed either a long research of use of 14C in archaeological contexts^34^ and/or have been the recent focus of high-resolution sampling^35^.

We see a similar pattern across the continental US: the risk analysis highlights the intermountain west and southwestern plains as being very well-sampled, as well as Michigan, New England, and a small section of the Oregon coast. Other portions of the United States, including the American Bottom, the coastal plains of Texas, northern California, the western Great Basin, Washington, southern Arizona, are all significantly undersampled relative to the number of dated sites. Researchers studying patterns in radiocarbon data should therefore be concerned about doing country-wide analyses that include areas with both high and low risk/density ratios.

### Case studies comparing number of sites and dates

We also conducted regional case studies where we compared the number of sites within a region to the number of radiocarbon dates within a region (sensu^36^). These graphs demonstrate radiocarbon sampling within a region: as the number of recorded sites increase, the number of radiocarbon dates should also increase. We fit a standardized major axis line to account for the sampling error not just on the y-axis (number of dates), but also on the x-axis (number of sites). The slope of the line informs the average number of radiocarbon dates per site within the region.

**Fig. 5:**
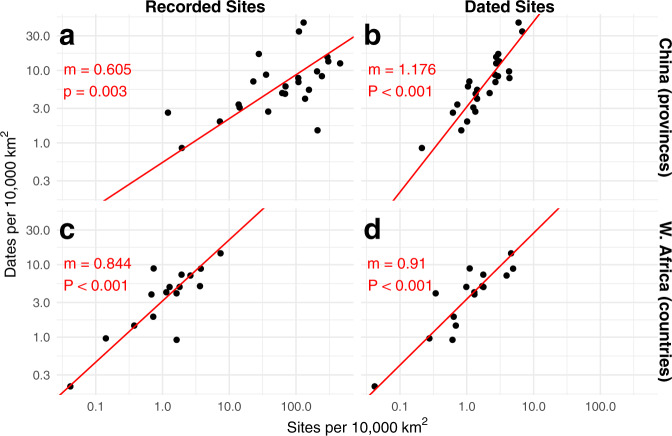
The density of sites versus dates for China (provinces) and Western Africa (countries). Each plot is log-log transformed. The relationship between the spatial density of recorded archaeological sites and dates across each region is sub-linear, indicating that enhanced recording of archaeological resources does not produce a higher density of dates. China shows a super-linear relationship between the density of dated sites and dates, suggesting over-sampling of dates in provinces with higher densities of dated sites; this relationship in Western Africa remains slightly sub-linear.

**Fig. 6:**
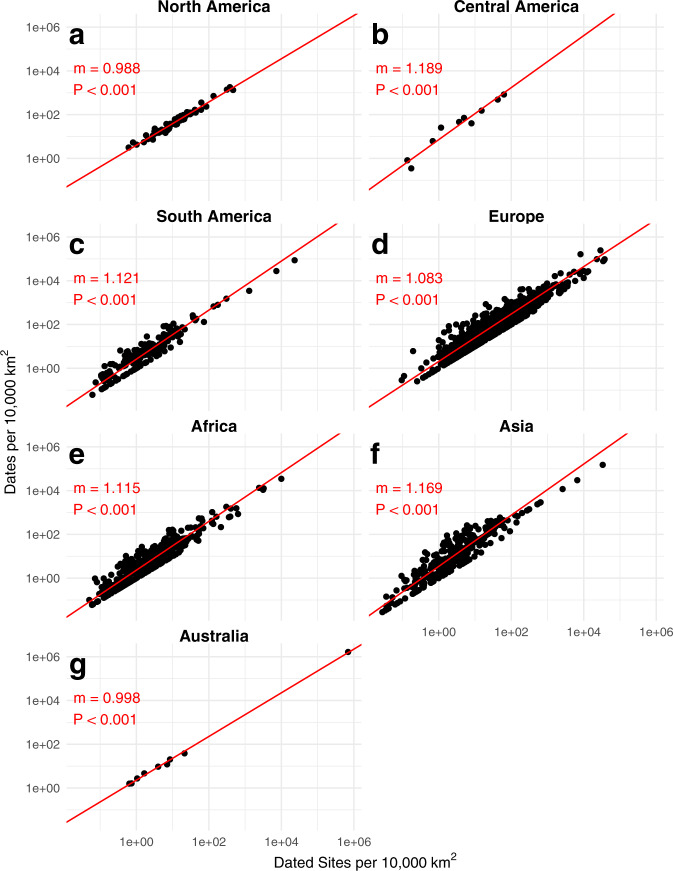
The density of dated sites versus dates at continental scale. Each point represents an Administrative Level 1 region (state/province) within the continent. Each plot is log-log transformed. North America and Australia demonstrate an effectively linear relationship between dated site density and date density, while Central America, South America, Europe, Africa, and Asia have super-linear relationships such that regions with a higher density of dated sites have an enhanced number of dates for those sites.

**Fig. 7:**
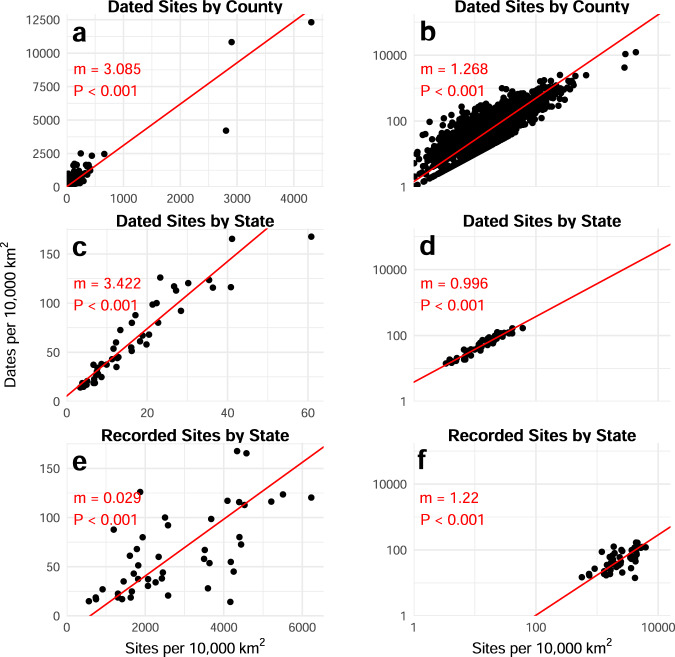
Focused case study of data in the United States, graphed linearly (left column) or logarithmically (right column). (**a**,**b**) Dated sites by county; (**c**,**d**) Dated sites by state; (**e**,**f**) Recorded Sites by state.

For all below quality assessments (Figs. 5–7), we divided the number of sites and dates by the area of the sampling units themselves to make them comparable. The expectation is that a representative sampling method would produce more radiocarbon dates per unit area as the number of sites per unit area increases. Given the variability in how “archaeological site” may be defined across the globe, this expectation may not hold globally, but it should hold for regions with similar site identification methodologies and sampling intensities.

### Regional case studies

For two case studies, we had site counts from other sources: China by province^37^ and Western Africa by country^38^ (Fig. 5a,b). By graphing the geographic density of radiocarbon dates against the geographic density of sites within a given region, we can identify the representativeness of radiocarbon dating in a given area, as well as outliers that have been over- or under-dated relative to the rest of the provinces or countries in the area. These graphs help identify whether the data can be considered representative of the archaeological record of the region that they cover.

Since many places in the world do not have site count data, we provide an alternative to test whether dates are considered representative. We graphed the number of radiocarbon dates against the number of sites that have been radiocarbon dated, generated from the radiocarbon database itself (Figs. 5c,d, 6a–g). Given how these data are generated, the p-value is impacted by autocorrelation and therefore of minimal value. Slopes greater than 1 (Figs. 5b, 6b–f) demonstrate the regions with a higher density of dated sites have a disproportionately high density of radiocarbon dates, while a slope of less than 1 (Fig. 5d) demonstrate that regions with a lower density of sites have a proportionally higher density of archaeological dates (though the slope in 5d is greatly influenced by abnormally-low site density in Guinea). Slopes closer to (Fig. 6a,g) demonstrate that the archaeological sites are representatively sampled, even if they may not be well sampled.

### United states case studies

Given the size of the United States dataset and the relatively consistent (compared to global methodology) site reporting measurements, we have conducted several comparisons between the site count and date count both by US state and county.

We conducted similar analyses as above: we compared the geographical density of dated archaeological sites to the density of radiocarbon dates, at both the county (Fig. 7a,b) and state (Fig. 7c,d) scale. We also compared the density of recorded archaeological sites per state (acquired from state files, SHPOs, historical commissions, etc., with code available in the *p3k14c* R package) against the density of radiocarbon dates per state (Fig. 7e,f). We plot these data on both raw and log-log scales in order to demonstrate the effect of outliers and test for non-linearity among the regions. All graphs show a clear positive relationship between the number of sites and the number of dates. Outliers have a clear effect on the linear scale (Fig. 7a,c). Notably, the three county outliers are some of the smallest counties for which we have data (Fairfax, VA, and Salem, VA are the smallest, while Nantucket, MA is the 7th smallest).

This shows the importance of scale when considering sampling: some states and counties are better sampled for both sites and radiocarbon dating than others, but radiocarbon sampling methodologies between states and counties are consistent.

## Usage Notes

For general radiocarbon data use, we recommend choosing data appropriate to the research question. As mentioned previously, some of the older data may require verification to ensure the link between humans and the dated object is secure. We have included region-specific usage and quality notes below to aid data usage.

### Regional usage and quality

For the following Regions, where one of the co-authors is considered a specialist, we have included a brief description of the regional dataset. We do not feature specialists for Oceania, South Asia, Southeast Asia, or North Asia. Northern South America is also underrepresented by our co-author specialities, though many continent-wide datasets include this area. We have chosen not to write as though we are experts for these regions, and we encourage readers to look directly at region-specific studies for these particular areas for recommendations.

#### Africa

P3k14c contains 11,129 radiocarbon ages for all of Africa. Prior to p3k14c, as elsewhere in the world, radiocarbon data for Africa were accessible across several regional databases compiled by distinct research teams with differing research goals. The majority of African data presented here are aggregated from eight sources, available online or on request, with a small number of dates available from other sources.

While there are frequent overlaps between databases, large parts of the continent are entirely unrepresented in these sources or feature only selective, project-specific data compilations. Many of the contributing datasets have research foci on the Holocene collected for a subregion of the continent. Kay *et al*.^38^ present c. 3000 Holocene archaeological dates to investigate land-use changes associated with food production across West and Central Africa, while the complementary “aDRAC” online repository archives c. 1500 Holocene Central African dates^39^. From Saharan and north Africa, Manning and Timpson^40^ collated >3000 Holocene dates to investigate demographic responses to climate shifts. Further Holocene dates for the region are recorded in the online MedAfriCarbon database of Lucarini *et al*.^41^. The CalPal database, maintained and distributed by Weninger^42^, archives c. 1100 additional Holocene dates for north-east and north-west Africa.

Regarding underrepresented regions, Eastern Africa is currently one of the less well-represented regions in the database, with only several hundred dates spanning thousands of years^43^. For parts of southern Africa, an online database integrated with OxCal presents c. 2500 dates spanning the entire radiocarbon age range^44^. As evident in a map of site distributions, there are few data for much of southern Africa outside of South Africa. Meanwhile, thirteen African countries (Madagascar, Zambia, Cape Verde, Comoros, Ivory Coast, Djibouti, Eritrea, Guinea-Bissau, Malawi, Mauritius, Sao Tome and Principe, Seychelles, South Sudan) are entirely unrepresented in the p3k14c compilation, and a further six countries, including Ethiopia, record less than ten radiocarbon dates each (also Somalia, Sierra Leone, Liberia, Guinea, and the Gambia). These patterns likely reflect both the remit of the published databases, and the true incidence of radiocarbon dates in these countries. Given the differing cut-off age ranges utilised in the different contributory data compilations, inter-regional comparisons beyond the Holocene are likely to be invalid.

Although more comprehensive regional data collations are in progress for Africa, we recommend against conducting continent-wide analyses of radiocarbon data given dramatically different sampling strategies throughout the continent at this juncture.

#### Southwest Asia

P3k14c contains a total of 6,222 radiocarbon dates in Southwest Asia collected from many regional databases^42,45–53^. Unlike other areas of the world where archaeological work is routinely integrated into planning and construction industries given a longer tradition of commercial archaeology (e.g., North America, central and northern Europe), in southwestern Asia most archaeological investigations are carried out by academic projects. As a consequence, this region is prone to spatial and chronological biases due to investigator bias.

Regarding chronological biases, for later historical periods, archaeologists rely more on short-lived pottery types and historical media (e.g., clay tablets) for dating archaeological layers rather than using radiometric dating. A certain reluctance in using radiocarbon dating among those archaeologists digging Iron Age sites is also justified by the Hallstatt radiocarbon calibration plateau (ca. 2750–2350 cal BP) which makes it difficult to obtain refined radiocarbon-based chronologies. On a pan-regional scale, the present dataset guarantees a good chronological coverage prior to ~3000 cal BP, while other regions such as Mesopotamia, Anatolia and Iran show a research-biased drop in the available radiocarbon dates from ∼4000-3500 cal BP onwards as shown recently by^54^.

Regarding spatial bias, 40% of the radiocarbon dates come from the Southern Levant (modern Israel and West Bank). This is due to the higher research budget of Israeli archaeological teams interested in producing or improving an absolute chronology. Additionally, certain chronological periods are more likely to be sampled than others. A clear research bias in the southern Levant is due to the interest of many archaeologists in providing a better chronology for the Early Bronze Age sub-periods (ca. 3800 - 2500 BC) and the Late Bronze Age/Iron Age transition (ca. 1200 - 950 BC)^55–60^.

#### East Asia

P3k14c has 5,818 radiocarbon ages for East Asia (China, Japan, and Korea). There are no comprehensive radiocarbon databases for the area. Most of the data come from mainland China (4,285), many come from Japan (1,433), and few come from Taiwan or South Korea (3). There are only three contributing data sources for this region^17,61,62^.

For China, the Chinese Academy of Social Sciences publishes compilations of all radiocarbon dates on roughly 30 year intervals^63^. The dataset we present here relied on dates that were compiled by^61^ for a meta-analysis of settlement site density in the People’s Republic of China, which include datasets from the Chinese Academy of Social Sciences. Regarding scope, the earliest dates in this database range as early as 40,000 BP. We also worked with data from a second database which focused on radiocarbon dates carried out directly on or associated with the introduction of crop remains^17^. The earliest dates in this database date to roughly 9000 BP. Because of its focus on association with published crop remains, this database does not present a comprehensive view of radiocarbon dates across East Asia. The dataset we present here should thus represent a relatively comprehensive set of dates from the People’s Republic of China, which is our area of expertise and which has been the focus of previous metadata compilations. Missing dates from the People’s Republic of China should only represent dates carried out over the past 8 years.

For Japan, ^64^ gathered data from eastern Japan to study Jomon demography between 7,000 and 3,000 BP. The uncalibrated data therefore range from 7,430 to 2,500 14C BP. The authors only gathered radiocarbon ages dated with the more accurate Accelerated Mass Spectrometer (AMS) method, allowed for only a narrow δ^13^C range, and removed all marine samples. The data are therefore very concentrated in three provinces of Japan for a fairly narrow temporal range.

The dataset we present here is sorely lacking comprehensive data from Taiwan, the rest of Japan, and Korea. Additionally, Southeast Asia, Central Asia and South Asia are all lacking in radiocarbon data as these regions fell outside of our region of expertise and there are no preexisting datasets currently in these regions.

#### Europe

P3k14c has 77,393 radiocarbon dates from Europe. Despite having been the focus of numerous regional and pan-regional surveys, there is currently no single comprehensive database for 14 C measurements. The p3k14c dataset collates all major existing resources, supplemented by specific regional assessment of the published and unpublished grey literature provided by several of the co-authors^41,42,45,47,48,65–73^.

Temporally speaking, the vast majority of dates are 2000 calBP or younger, primarily from three distinct resources covering Norway, Finland, and the UK and Ireland. Though this does not entirely reflect the variety of local research traditions, it does emphasise that, in many respects, the collation of 14 C dates has been largely driven by studies focusing on Prehistoric, and especially Later Prehistoric (Mesolithic, Neolithic and to a lesser extent Bronze Age) periods. From a spatial point of view, the European dataset is characterised by a marked discrepancies in terms of number of dates and sites per country, but also in terms of average number of dates per site (see above KDE analysis). The latter trend results from the recent multiplication of studies combining extensive sampling and Bayesian statistics. While such research arguably provides exceptional records at site-level, they also lead to major regional imbalances where a minority of intensively dated sites co-exists with a majority of limitedly investigated sites, thus hampering regional contextualisation (e.g.^74^).

Given the long history of research and early use of 14C in Europe, pre-existing compilations often extensively overlap in content, although the recordation of site name, cultural attributions, and locational data sometimes differ significantly across resources. In many instances, it is frequently difficult to identify qualitative criteria linked to database compilation and management (e.g.^67^), though recent efforts to improve and share auditing methods are noticeable^75,76^. As a result, the available raw data contain redundant and/or conflicting information, which was tackled here through a combination of automated and qualitative assessment of the evidence (see above).

#### North America

P3k14c contains 64,933 radiocarbon ages from North America divided between the USA (56,612) and Canada (8,322). We have included all archaeological data from the Canadian Archaeological Radiocarbon Database (CARD) and supplemented it with data collected through the NSF-funded project *Populating a Radiocarbon Database of North America* (PI: Robert L. Kelly), which compiled data from the lower 48 United States. The UWyo2021 dataset benefitted from several existing albeit smaller collection efforts whose results were generously shared, and through a number of state radiocarbon databases^77^. They also searched for dates through open Google searches, searches (manual and digital) through journals, including all the state journals and bulletins they could obtain, and through searches of SHPO records where possible. Data from CARD are submitted voluntarily by researchers and have a minimal review process. This region contains no cutoff date for age, and they recommend that researchers conducting research on earlier time periods personally verify the earlier dates.

In the USA, the number of archaeological dates by region and state differs widely, from >12,500 for California to <225 for New Hampshire, reflecting not just state sizes but also research and CRM intensity. Comparing the number of dates to the number of sites recorded in SHPO files, some states (California, Texas, Wyoming, Pennsylvania, Washington, Florida, Oregon, Ohio, Illinois) are better sampled than other states, while others (Arkansas, Idaho, South Carolina, North Carolina) are relatively under-sampled, although not poorly sampled. Differences are due to uneven access to unpublished data and grey literature. The dates may also suffer from investigator bias (e.g., a focus on research surrounding the adoption and spread of agriculture).

The data from the United States and Canada have obfuscated locational information (per the section on the Obfuscation of precise coordinates for certain dates), which are freely available through tdar, github, and zenodo. Data with more precise locational information are also available through tdar under restricted access. The UWyo2021 dataset only provided county-centroid locations, however. Should researchers require more precise locations than county/division centroids, the data may still be obtained by reaching out to the State Historic Preservation Office (SHPO).

#### Central America

P3k14c contains 1,218 radiocarbon ages from Central America. Until recently, radiocarbon dates published at sites across Mesoamerica have not been compiled and organized in any comprehensive manner. Two recent dates-as-data studies^78,79^ identified published dates from the literature of the Maya lowlands, in efforts to identify social and political developments associated with climatic change. These studies, and subsequent compilations of published 14C dates, has led to the creation of the Mesoamerican Radiocarbon Database (MesoRAD)^80^, which is the only dataset contributing to p3k14c’s Central American sample. MesoRAD represents the largest compilation of published data from the Archaic to the Colonial periods in Mesoamerica.

Chronologically, all dates identified in the literature were included in the database, with the earliest secure dates as early as 9785-9290 cal BCE and the most recent associated with modern landscape disturbance (e.g., plowing and burning). Despite taphonomy and time depth, data show good coverage for the Preclassic/Formative period (1200 cal BCE to 300 CE), due to research agendas focused on timing for the origins of village life across the region (e.g.^79,81–83^). An increase in dates is noted from the Early Classic periods (300 CE to 600 CE) to the Late Classic period (600 to 750 CE), associated with large-scale population increase recorded across the region. Similarly, a drop in the frequency of 14 C dates can be identified in the transition from the Late Classic to Terminal Classic period (~750 CE), concomitant with identified reductions in populations associated with what is commonly described as the ‘Classic Maya collapse’. Several well-dated Postclassic sites, such as Mayapan^84^ represent the largest concentrations of Postclassic dates in the sample, while other regions, such as northern Belize and the Peten Lakes, include lower numbers of Postclassic dates. Finally, there are fewer dates associated with contexts after European conquest.

Spatially, large gaps continue to exist where no 14C dates have yet been compiled, including central Mexico, Oaxaca, and the Gulf Coast region, as well as other parts of Mesoamerica and Central America.

#### South America

P3k14c contains 7,668 radiocarbon ages from South America. Currently there does not exist a comprehensive radiocarbon database for the entire continent, but there are a few databases that have been compiled with very specific temporal and spatial constraints and goals. The South American data is derived from existing databases (e.g.^85–90^ and papers where a large number of dates are published (e.g.^91–93^). We are aware that many of these datasets are incomplete and largely outdated, particularly because South America has witnessed a surge in radiocarbon dating over the last two decades but also because initial compilations of radiocarbon dates were regionally and temporarily biased.

Regarding temporal biases, this dataset does not include dates older than 15,000 BP and we remain skeptical of human occupations dating earlier than this date. A recent study^93^ includes a recent continent-wide review of late Pleistocene and early Holocene dates for timing the occupation of South America and include older dates as well as cautionary notes on their use. Regarding recent dates, we do not include a cut-off point, but many of the databases we rely on did not have dates younger than 2000 BP. Furthermore, many researchers working in South America typically do not use radiocarbon dating on materials that post-date the European conquest, approximately 500 years ago^86^. Therefore, the late Holocene record is limited in coverage.

We recognize the imperfect quality of the coverage of this dataset. We are more certain about omissions from regions of our general expertise (including central Argentina, Bolivia, Chile, southern Peru, and southeastern Brazil), and recognize that northern South America might be poorly covered and could incorporate significant oversights. For Bolivia, because many of these include roughly the same dates as well as various errors, we have relied on^94^ for a countrywide review of radiocarbon dates for updated and corrected information about site location, lab codes, dates, etc.

Finally, we are in the process of updating a new synthetic continental scale database based on primary literature, but this work is still in process and for researchers interested in a broad sweep of data, this database might be suitable.

### Usage notes for *R* users

We provide an *R* package called *p3k14c* to facilitate access to the scrubbed/fuzzed dataset for *R* users, and to make the quality analysis, table and figure generation code available (see the Code Availability section below). The *R* package is available as a Github repository (https://github.com/people3k/p3k14c), and the version of the package used that created the analyses reported here are archived on Zenodo^95^. Users interested in accessing the p3k14c data should refer to the package documentation in R, and to the README.md file available in the Github repository.

#### *Python* code for scrubbing radiocarbon data

We provide in full the suite of Python 3.7 scripts used to process the dataset as of the time of submission. The code is hosted under version control on GitHub (https://github.com/people3k/p3k14c-data-scrubbing), also archived on Zenodo^96^. This suite contains complete replication steps, usage instructions, and structure explanations as part of its **README.md**. Further, all blocks of code are paired with commented documentation explaining their function should the user desire to modify the programs or obtain a finer-grained understanding of the suite.

The suite consists of three main scripts. The primary script, **scrub.py**, accepts unprocessed radiocarbon records and performs the scrubbing procedures specified in prior sections. This script relies on **removeDuplicates.py**, the duplicate-handling division of routines, which is also capable of being run independently if the user desires only to handle a dataset’s duplicates without scrubbing it first. Further, **fuzz/fuzz.py** converts all US, Canada, and GuedesBocinsky2018 spatial coordinates to county centroids, province centroids, and truncated coordinates, respectively. Lastly, the scripts used to perform the unicode character correction procedure are located in the **charfix** directory. An anaconda **environment.yml** is specified for ease of consistent environment creation with proper package versioning.

#### *R* code for quality analyses

A research compendium, complete with R code, to run the quality analyses and produce the figures and tables presented here is available as a part of the *p3k14c* R package on Github (https://github.com/people3k/p3k14c) and archived on Zenodo^95^. Code was run in RStudio version 4.0.5; other details on the runtime environment are available in the colophon of the research compendium. This package also includes the scrubbed/fuzzed data, site count data, and an executable paper that recreates the figures and tables in this publication.

## Data Availability

Code used to prepare the datasets and for the data quality analyses reported above were developed using both the *R* and *Python* computing languages.
